# Wheat germ oil enrichment in broiler feed with α-lipoic acid to enhance the antioxidant potential and lipid stability of meat

**DOI:** 10.1186/1476-511X-12-164

**Published:** 2013-11-04

**Authors:** Muhammad Sajid Arshad, Faqir Muhammad Anjum, Muhammad Issa Khan, Muhammad Shahid, Saeed Akhtar, Muhammad Sohaib

**Affiliations:** 1 National Institute of Food Science and Technology, University of Agriculture, Faisalabad, Pakistan; 2 Department of Chemistry and Biochemistry, University of Agriculture, Faisalabad, Pakistan; 3 Department of Food Science and Technology, Bahauddin Zakariya University, Multan, Pakistan

**Keywords:** α-Lipoic acid, α-Tocopherol, Wheat germ oil, Total phenolic contents, DPPH, Frap

## Abstract

**Background:**

Lipid peroxidation is the cause of declining the meat quality. Natural antioxidants plays a vital role in enhancing the stability and quality of meat. The supplementation of natural antioxidants in feed decreases lipid peroxidation and improves the stability of meat.

**Methods:**

The present research was conducted to determine the effect of α-lipoic acid, α-tocopherol and wheat germ oil on the status of antioxidants, quality and lipid stability of broiler meat. One day old male broilers were fed with different feeds containing antioxidants i.e. natural (wheat germ oil) and synthetic α-tocopherol and α-lipoic acid during the two experimental years.

**Results:**

The feed treatments have significant variation on the body weight and feed conversion ratio (FCR) while having no influence on the feed intake. The broilers fed on wheat germ oil (natural α-tocopherol) gained maximum body weight (2451.97 g & 2466.07 g) in the experimental years 2010–11 & 2011–12, respectively. The higher total phenolic contents were found in the broilers fed on wheat germ oil plus α-lipoic acid in breast (162.73±4.8 mg Gallic acid equivalent/100 g & 162.18±4.5 mg Gallic acid equivalent/100 g) and leg (149.67±3.3 mg Gallic acid equivalent/100 g & 146.07±3.2 mg Gallic acid equivalent/100 g) meat during both experimental years. Similar trend was observed for the 2,2-diphenyl-1-picrylhydrazyl (DPPH) and ferric reducing antioxidant power assay (FRAP). The production of malondialdehydes in the breast and leg meat increased with progressive increase in the time period. The deposition of α-tocopherol (AT) and α-lipoic acid (ALA) contents were found to be higher in the broilers fed on wheat germ oil plus α-lipoic acid in breast and leg meat during the both experimental years.

**Conclusion:**

In conclusion, the combination of wheat germ oil and α-lipoic acid has more beneficial for stability and the quality of the broiler meat and more work should be needed in future for the bio-evaluation of this kind of functional meat in humans.

## Introduction

The lipid oxidation in poultry meat is one of the primary causes for deterioration in muscle quality, apart from microbial spoilage [1]. The use of antioxidants is one the most important ways for lipid oxidation prevention because antioxidants restrict access of oxygen and retards the development of off-flavors and improve oxidative stability of the meat and meat products [2]. The synthetic antioxidants such as butylatedhydroxytoluene (BHT) and butylatedhydroxyanisole (BHA) are being widely used to control lipid peroxidation in meat. But their use is not safe so the use of natural antioxidant in meat products is a promising tool to enhance the shelf life of meat [3].

The administration of alpha-lipoic acid through feed decreases oxidative stress and other antioxidants restore their reduce levels of *in vivo*. Due to its biochemical characteristics, α-lipoic acid is considered as a potential source of therapeutic for the redox-unbalanced diseases and the treatment of energy impaired. The α-lipoic acid is found in a widespread variety of foods for plants and animals origin, mainly in kidney, heart and liver meats as well as spinach, broccoli and potatoes [4]. Alpha-lipoic acid can also be synthesized by de novo, so not considered as a vitamin [5]. Alpha-lipoic acid has the ability to directly scavenge reactive oxygen species (ROS) and has potential to regenerate endogenous antioxidants such as vitamin C, glutathione reduced vitamins E and also has metal chelating potential resulting in reduced ROS production [6]. The α-lipoic acid and α-tocopherol enriched broiler meat increases the oxidative stability and antioxidant potential of the broiler meat and also responsible for decreasing the TBARS (Thiobarbituric acid reactive oxygen species) content in broiler meat where (150 mg α-lipoic acid/kg) supplemented feed [7].

Alpha-tocopherol (Vitamin E) is a suitable supplement and added into animal diets as a highly effective lipid-soluble and chain breaking antioxidants [8]. Unlike other fat-soluble vitamins, α-tocopherol has not been reported to be accumulated to toxic levels. The α-tocopherol accumulation at tissue levels are strongly regulated via increased hepatic metabolism and excretion that could, theoretically, alter environmental toxins, metabolism of drugs, and other nutrients [9]. The feed supplementation with α-tocopherol has shown the most effective means of improving the oxidative stability of meat lipids and inhibiting oxidation of cholesterol and possesses 15 times more powerful antioxidant properties [10]. The accumulation of vitamin E in tissues has been related to dietary supplementation levels during pre-slaughter period in broilers [11].

The wheat germ oil (WGO) consists of about linoleic acid (18:2 n6) (56%), which is an essential fatty acid [12]. Total unsaturated and polyunsaturated fatty acid (PUFA) contents of WGO are about 81 and 64%, respectively. The wheat germ oil possesses various biological properties as anticancer and antioxidant agents [13]. Wheat germ oil due to high concentration of bioactive compounds has also a number of nutritional and health benefits such as reduction in plasma and liver cholesterol levels, improving physical endurance/fitness, and possibly delaying effects of aging. In Pakistan, cardiovascular complications are consider the major cause to the mortality of people and this disease is enhanced due to high level of cholesterol.

## Materials and methods

### Procurement of raw material

The wheat germ was collected from Sunny Flour Mills, Lahore, Pakistan. Alpha lipoic acid and synthetic alpha-tocopherol were purchased from Shaanxi Sciphar Hi-Tech Industry Co, Ltd China and from Merck (Merck K Ga A, Darmsladt, Germany) respectively. All the reagents and chemicals for this research were purchased from Sigma Aldrich (Germany) and Merck (Germany). One day old male 180 chicks (50±5 g body weight) were purchased from Jadeed Chicks Private Limited, Faisalabad, Pakistan. Fine saw dust (for the bed preparation of the broiler chicks) and Bromo-Sept (for the disinfection of the pens in the student reserved research room) were procured from the local market of Faisalabad, Pakistan.

### Extraction of wheat germ oil

The wheat germ oil was extracted through solvent extraction technique by using the facilities available at Pakistan Council of Scientific and Industrial Research (PCSIR) Laboratories Complex, Lahore, Pakistan. The wheat germ was taken in a soxhlet extraction apparatus and extracted with the use of commercial hexane for 10 h. Then, solution was filtered followed by solvent removal in a rotary evaporator under vacuum at 40°C. The yield of the oil was recorded as 12%. The wheat germ oil was kept at 5°C in a refrigerator.

### Experimental plan

The feed of chicks was supplemented with alpha-lipoic acid, synthetic alpha-tocopherol and wheat germ oil. One day old broiler chicks were used for this experiment. The broilers were divided in to 6 groups with 3 replicates in each group. Each replicate comprise of 10 birds. Two trials were conducted for this study. One hundred and eighty birds purchased for each trial. First trial was conducted during the year 2010–11 and the second trial conducted during the year 2011–12. The composition of basal diet consists of Corn (39 g), Rice broken (2.07 g), Rice polishing (5.60 g), Cotton seed meal (2.20 g), Canola meal (2.0 g), Corn gluten 60% (2.30 g), Sunflower meal (12.40 g), soybean meal (15 g), Fish meal (6.60 g), L-lysine (0.15 g), DL-methionine (0.08 g), Dicalcium phosphate (1.20 g), Limestone (0.90 g) and Premix (0.50 g) which contains the following (Vitamin K3: 0.02 g, Vitamin B1: 0.02 g, Vitamin B2: 0.08 g, Vitamin B6: 0.1 g, Vitamin B12: 0.05 g, Niacin: 0.12 g, Calcium Pantothenate: 0.06 g, Folic Acid: 0.02 g, Biotin: 0.03 g), Nutrient composition (calculated): Metabolized energy (2900 Kcal/kg), Crude protein (21.03%), Lysine (1.1%), Methionine (0.52%). The composition of WGO consists of linoleic acid (53%) and 77% total unsaturated and polyunsaturated fatty acid contents in the wheat germ oil was about 61%. The α-tocopherol contents in the wheat germ oil was about (166.66 mg/100 g).The feed supplementation plan is described in the Table 1.

**Table 1:** Broiler feed supplementation

	**Supplementation per kg feed**
**T**_**1**_	Control
**T**_**2**_	Wheat germ oil (200 mg natural α-tocopherol)
**T**_**3**_	Synthetic α-tocopherol (200 mg)
**T**_**4**_	α-Lipoic acid (150 mg)
**T**_**5**_	α-Lipoic acid (150 mg) + Wheat germ oil (200 mg natural α-tocopherol)
**T**_**6**_	α-Lipoic acid (150 mg) + Synthetic α-tocopherol (200 mg)

Natural α-tocopherol was used from wheat germ oil. After the quantification α-tocopherol from wheat germ oil, the wheat germ oil was used as the natural source of α-tocopherol by competing natural α-tocopherol equivalent to syntheticα-tocopherol.

### Experimental site management and conditions

The research room and all the pens were thoroughly white washed. Bromo-sept and formalin aqueous solutions with ratio 1:12 was used to disinfect inside and outside the pens prior to initiation of the trial. A 2–3 inch thick layer of saw dust was made in each pen as litter to keep the pen bed dry and soft. All the drinkers and feeders were washed thoroughly with water and disinfected with Bromo-Sept aqueous solution in the ratio of 1:3. All the pens were tagged with their respective treatments and replication numbers. The birds were kept in white washed reserved research room having 20 pens of 12 sq feet capacity each. A layer of saw dust was used as litter in each pen that was stirred regularly during the experiment to keep it in dry condition. The temperature of the experimental room was maintained at 35°C during the first week. The temperature was then lowered by 5°C till it reached to 25°C ±2. Twenty four hour light and proper ventilation were maintained in the experimental room throughout the experimental period. The feed and fresh water was given to experimental bird’s ad-libitum.

### Chick’s vaccination

A glucose solution (50 grams per 5 Liter) was given to the chicks after 1–2 hours of distribution in pens for the removal of waste from the body of chicks. At the age of 2 days a solution of Cotrim-50 (1 gram per 5 Liters of water) was given to the chicks for prevention from bacterial infections. Chicks were vaccinated with N.D+I.B at the age of 3–4 days for the prevention from New Castle Disease and wild cough. At the age of 10 and 18 days birds were vaccinated with Gamboro vaccine for the prevention of Gamboro disease. At the age of 24 days birds were vaccinated with Lasuta vaccine for the prevention of New Castle Disease.

### FCR calculation

The chicks were weighed on first day of trial. The weights of the birds were carried out on weekly basis for six weeks to calculate the weight gain during each week. The mortality was also recorded. At the start of each week a measured quantity of feed was fixed in each pen for feeding the chicks. At the end of each week feed conversion ratio was calculated by dividing the feed consumed in the whole week with weight gain by the bird in the respective week by the formula given below.

$$\begin{array}{l} \mathrm{FCR}=\mathrm{Feed}\;\mathrm{consumed}\;\mathrm{by}\;\mathrm{the}\;\mathrm{bird}\;\mathrm{in}\;\mathrm{one}\;\mathrm{week}\\ \;\;/\mathrm{weight}\;\mathrm{gain}\;\mathrm{by}\;\mathrm{the}\;\mathrm{bird}\;\mathrm{in}\;\mathrm{one}\;\mathrm{week} \end{array}$$

### Slaughtering of the experimental birds

The experiment was conducted for 42 days. After the expiry of experiment, the birds were slaughtered according to the Halal Ethical guidelines. After slaughtering, breast and thigh of broilers were deboned, wrapped with aluminum foil and finally packed in polythene zip lock bags. The samples were stored in the freezer at −18°C for further analysis.

### Homogenization

The meat sample (5 g) was taken in 50 ml polypropylene tube having a cap and sample homogenized by using phosphate buffer and glycerol (20%) pH (7.4) by using homogenizer. The tubes were given rest in the ice cold water during homogenization. Filtration process was carried out to remove connective tissues from sample by using muslin cloth.

### Antioxidant contents in raw meat

The antioxidant contents in raw leg and breast broiler homogenized meat sample were estimated by the following analytical methods as described below.

### Total phenolic contents

The total phenolic contents in leg and breast meat was determined by following the method described by [14]. The homogenized meat sample (100 μL) was mixed with 95% ethanol (500 μL), distilled water (2.5 mL), and 50% Folin-Ciocalteu reagent (250 μL). After 5 min, 5% Na_2_CO_3_ (500 μL) was added to the resultant mixture, which was vortexed and placed in the dark room for 1 h. The absorbance of the sample was recorded by running sample through spectrophotometer (CESIL CE7200, England) at wave length 725 nm. Total phenolic contents were measured as Gallic acid equivalent ranges from 0 to 450 μg/mL.

### Free radical scavenging activity (DPPH assay)

The leg and breast broiler meat samples were subjected to analysis of the DPPH radical scavenging activity according to the procedure outlined by [15]. The antioxidant activity of the homogenized meat samples were assessed by measuring their scavenging abilities to DPPH stable radicals. A sample of 125 μL was mixed with 0.0012 m DPPH solution followed by the addition of 95% MeOH up to a final volume of4mL and allowed to rest for 1 hour at room temperature. The absorbance of the resulting solution and the blank were recorded spectrophotometrically at 515 nm. The inhibition of free radicals by DPPH in percent (%) was calculated by using the following formula.

$$\mathrm{Inhibition}\left(\%\right)=100\times \left(A_{\mathrm{blank}}-A_{\mathrm{sample}}/A_{\mathrm{blank}}\right)$$

### Ferric reducing antioxidant power (FRAP)

The ferric reducing antioxidant power in the leg and breast broiler homogenized meat samples were estimated by following the method described by [16]. A 200 μL sample was mixed with 500 μL sodium phosphate buffer (0.2 M, pH 6.6) and 500 μL potassium ferri cyanide (1%), and the resultant mixture was incubated at 50°C for 20 min. After addition of 2.5 mL trichloroacetic acid [TCA, (10%)], the mixture was centrifuged at 2200×g for 10 min. The upper layer (500 μL) was mixed with 500 μL distilled water and 100 μL ferricchloride (0.1%), and the absorbance was measured at 700 nm using a spectrophotometer (CESIL CE7200, England).

### Oxidative stability test

The leg and breast homogenized meat samples were tested for thiobarbituric acid reactive species (TBARS) by the procedure mentioned by [17]. The peroxidative reaction was initiated by adding 700 μL of FeSO_4_ 7. H_2_O (0.1 M) and 200 μL ofH_2_O_2_ (0.1 M) in7 mL of membrane suspension and placed in a water bath (37°C). One ml sample from the reaction flask was withdrawn at 30 minutes intervals for a period of 120 minutes and added to a volume of solution consisting of thiobarbituric acid (0.4%) trichloroacetic acid (10%) and hydrochloric acid (0.25 N). The mixture was heated in a boiling water bath for 15 minutes and then cooled. After centrifugation, the absorbance of the supernatant was recorded on spectrophotometer at 532 nm, and the extent of membrane lipid peroxidation was calculated by using the formula.

$$\begin{array}{l} n-\mathrm{Mole}\;\mathrm{of}\;\mathrm{malondialdehydes}\\ \;=\frac{\left(\mathrm{Sample}\;\mathrm{absorbance}–\mathrm{blank}\right)\times \mathrm{Total}\;\mathrm{sample}\;\mathrm{vol}.}{0.000156\times 1000\left(\mathrm{per}\;\mathrm{mL}\right)} \end{array}$$

### Alpha-tocopherol contents

#### Sample preparation

The sample was prepared according the method described by [18]. The homogenized meat sample (500 μL) was taken, and 1.5 mL of urea (6 M) was added to dissolve the meat tissue. 0.5 mL of ascorbic acid (5%) was added in the reaction mixture for the stability of the meat tissue. Then 1 mL of urea (6 M) was also added. The tube was flushed withN_2_, the screw caps were tightened, and the mixture was vortexed for 12 min to dissolve the sample. One milliliter of 0.1 M sodium dodecyl sulfate (SDS) solution was added and vortexed for 1 min to disintegrate the meat tissue. For the deproteination and freeing of alpha-tocopherol, 4 mL of ethyl alcohol containing 1% pyrogallol was added in the mixture. There aftert, petroleum ether (10 mL) was added, and centrifuged the mixture at 5000×*g* for 5 min to facilitate the separation of phases. The solvent layer containing alpha-tocopherol was separated in the vial, and the pooled solvent was evaporated under nitrogen. Alpha-tocopherol contents were dissolved in the mobile phase (100% methanol). The sample was filtered by using an anspec 0.45 μm microfilter, centrifuged at 5000×*g* for 5 min so as to collect all of the filtrate, and then stored in the dark.

The mobile phase consists of methyl alcohol (HPLC grade); 100% methanol was prepared by filtering it through a typhlon filter assembly and then adjusted to HPLC. The standard of alpha-tocopherol was prepared by using Sigma-Aldrich packed standard 1 mg/mL α-tocopherol as stock solutions from which further dilutions were made in ranges of 10, 20, 50, and 100 μg/mL. Alpha-tocopherol was separated and quantified using HPLC (PerkinElmer, Series 200, USA) chromatographic system with 290 nm wavelength UV–vis detector. The HPLC chromatograms were obtained by using a (C18) column, (250 mm × 4.6 mm, 5.0 μm), System controller SCL-10 A, water pump LC-10 AT, and flow controller valve FCV-10 AL with a mobile phase of 100% methanol at a flow rate of 1 mL/min.

### Alpha-lipoic acid contents

#### Sample preparation

The sample was prepared according the method described by [19] with some modifications. The sample (200 μL) was taken from each treatment in the glass tubes, then homogenized with 2 mL of 20% metaphosphoric acid (w/v) on ice, and extracted with 3 mL of hexane containing 250 μL of isoproponol by vortexing for 30 min. The sample was centrifuged at 1500×*g* and collected the upper hexane layer in a glass tube. This step was repeated twice and the hexane layer allowed drying with N_2_. The mobile phase was prepared by using acetonitrile and water (80:20(v/v)). The sample was eluted isocratically at a 1 mL/min flow rate. The standards of alpha-lipoic acid were prepared by dissolving alpha-lipoic acid in n-hexane as a stock solution (25 mg/mL), and further dilutions were made (20, 30, and 40 μg/mL, respectively). The alpha-lipoic acid was separated by injecting 20 μL of sample and quantified by using HPLC (PerkinElmer, Series 200, USA) chromatographic gradient system. The fluorescence detector was operated at excitation and emission wavelengths of 343 and 423 nm, respectively, with a CLC (C18) column(250 mm × 4.6 mm, 5.0 μm), system controller SCL-10A, water pump LC-10 AT, flow controller valve FCV-10AL, acetonitrile/water (80:20 v/v) mobile phase, and 1.0 mL/min flow rate.

### Statistical analysis

The data was statistically analyzed by Completely Randomize Design using three way analysis of variance (ANOVA) for the growth parameters broiler and two way ANOVA was done for the antioxidant parameters by using software (Statistic 8.1). The comparison of means was done by the Duncan Multiple Range test.

## Results and discussion

### Growth parameters

#### Body weight gain

**Figure 1:**
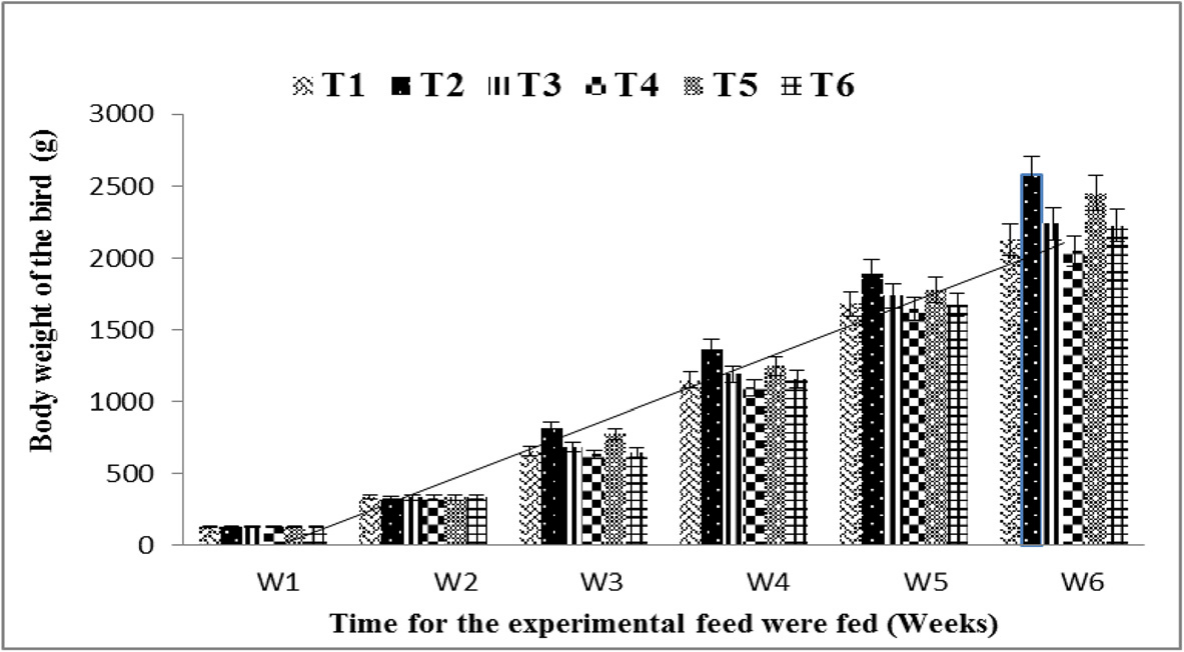
**Average body weight of broilers for two experimental years fed on different feeds.** Average body weight of two years study measured after every week after the Experimental feed were fed. Refer to Table 1 for the nature of each treatment.

The body weight gain of broilers at the start of the growth (1^st^ week) ranged between 126.12 to 133.71 g and 134.91 to 141.23 g during the experimental years 2010–11 and 2011–12, respectively. The body weight gain at the end of experiment i.e. 6^th^ week found to be ranged between 2087.09 to 2451.97 g and 2064.33 to 2466.07 g during the experimental years 2010–11 and 2011–12, respectively (Figure 1). The broilers fed on wheat germ oil supplemented feed (T_2_) exhibited significantly higher body weight gain at the end of the experiment (6^th^ week) followed by broilers fed on wheat germ oil plus α-lipoic acid (T_5_) in both the experimental years. However, the lowest body weight gain was recorded in broilers fed on α-lipoic acid (T_4_) during the experimental year 2010-11and 2011–12. The trend in body weight gain was observed identical in all treatments during both the experimental years. The body weight gain of broilers increased significantly with progressive increase in experimental weeks. The broilers fed on wheat germ oil (natural α-tocopherol) diet gained maximum weight during the experimentation but broilers fed on T_4_ (α-lipoic acid) gained the lowest body weight in both the experimental years. Since wheat germ oil possessed natural source of α-tocopherol which increased the body weight gain significantly. These findings are supported by Traber (1999) who described that natural α-tocopherol has 3 times higher biological activity than the synthetic α-tocopherol. Similarly the results are reported by [20] who found the highest body weight gain in the group with wheat germ enriched feed in rats. Some other scientists [21,22] have also reported enhanced weight gain and feed conversion efficiency in broilers receiving much higher (200 mg/kg) dietary concentrations of α-tocopherol [23].

The antioxidant supplementation in feed significantly improved the growth of broilers [24]. The decrease in body weight gain due to intake of α-lipoic acid by the birds is also reported by [25]. The findings of [7,26,27] have also indicated that the higher concentration of α-lipoic acid (150 mg) in the feed suppressed the growth performance of broilers. Recently [28] have also indicated that use of natural antioxidants is an effective way of getting the best result in terms of body weight gain in broiler birds. Shen et al. [29] reported that 0.5% and 1.0% α-lipoic acid supplementation for 3 weeks resulted in a decrease in final mouse body weight and carcass weights as the levels of dietary α -lipoic acid increased. They concluded that α-lipoic acid supplementation caused the significant decrease in carcass fat, which, in turn, decreased carcass weight. In the present investigation, the natural α-tocopherol yielded more body weight gain as compared to control whereas α-lipoic acid supplemented feed showed declining trend in body weight gain and these results are in consistent with the findings of scientists reported above. The average mortality of the broilers for two years of study was higher (10%) for broilers fed on wheat germ oil plus α-lipoic acid followed by for the broilers fed on wheat germ oil (8.3%), 6.6% for control, 6.6% for the broilers receiving feed with synthetic α-tocopherol, 6.6% for the broilers receiving synthetic α-tocopherl plus α-lipoic acid and 5% for the broilers fed on α-lipoic acid enriched feed.

### Feed intake

**Figure 2:**
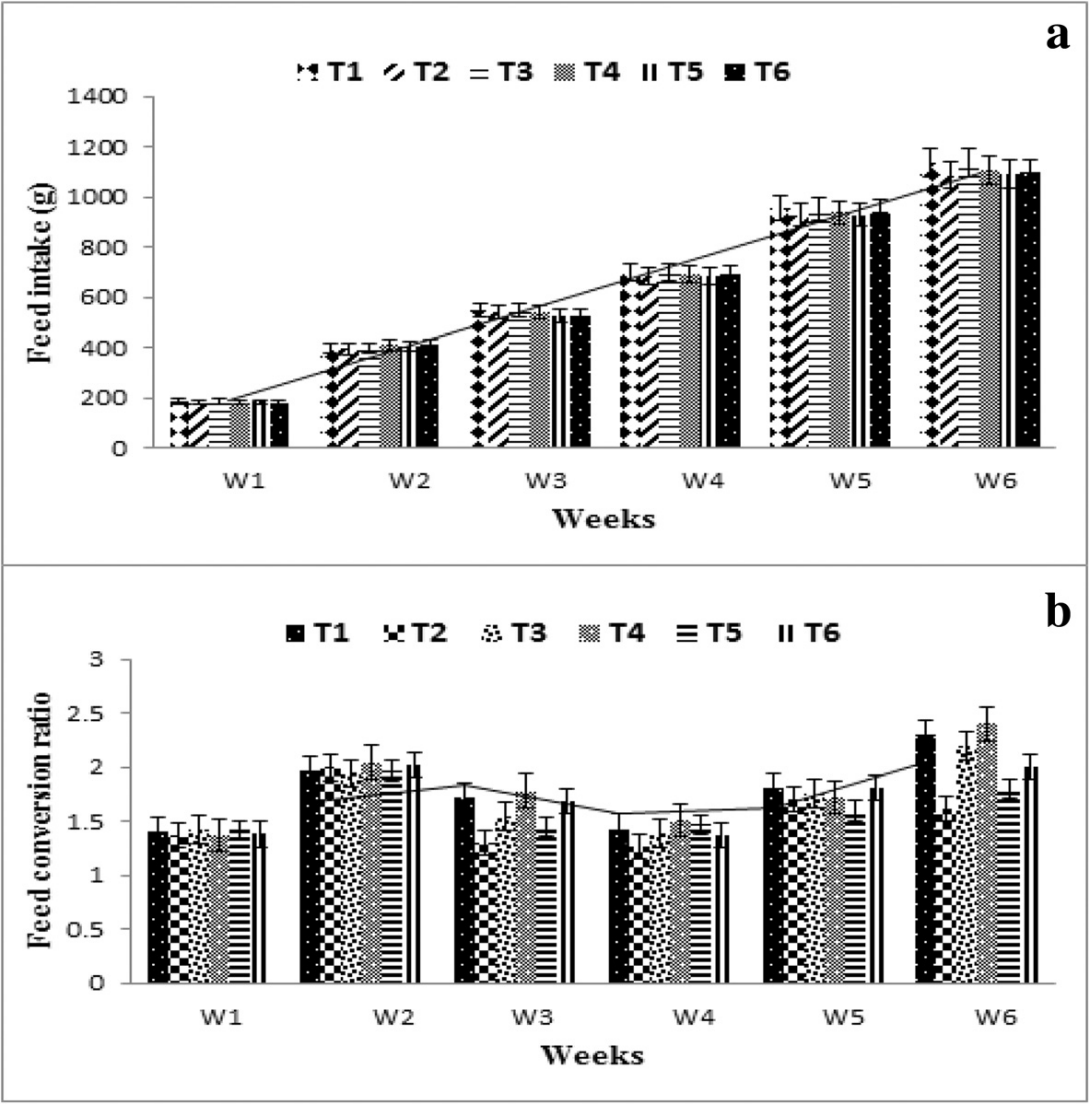
**Feed intake and feed conversion ratio of broilers fed on experimental feed. (a)**. Average feed intake of two years study measured after every week. **(b)**. Average feed conversion ratio of two years study calculated after every week. Refer to Table 1 for the nature of each treatment.

The feed intake of broilers ranged at the start of the experiment (1^st^ week) ranged between 162 to 169 g and 168 to 172 g during the experimental years 2010–11 and 2011–12, respectively however the feed intake at the end of the experiment (6^th^ week) ranged between 1114 to 1122 g and 1094 to 1115 g during the experimental years 2010–11 and 2011–12, respectively (Figure 2a). The results of the present study are in agreement with the findings of [30] who reported that supplementation of α-tocopherol did not influence the feed intake of the broilers. Rymer et al. [31] reported that the feed intake of broilers differed non-significantly by using the oil in the feed of broilers. Rezaeipour et al. [32] revealed that consumption of α-tocopherol enriched feed has no effect on the feed intake of broilers. The findings of these scientists support the results found in present study which also revealed non-significant difference due to treatments on feed intake.

### Feed conversion ratio (FCR)

The FCR of broilers during 1^st^ week ranged from 1.83 to 2.04 and 1.70 to 1.84 during the experimental years 2010–11 and 2011–12, respectively. The FCR of broilers at the termination of the experiment (6^th^ week) ranged between 1.64 to 2.01 and 1.57 to 2.06 during both experimental years i.e. 2010–11 and 2011–12, respectively (Figure 2b). The FCR (1.89) was found significantly higher in T_4_ (α-lipoic acid) followed by broilers fed on T_1_ (control) (1.84). The lowest FCR (1.65) was recorded in broilers receiving T_2_ (natural α-tocopherol) feed during the experimental year 2010–11. The FCR was found higher in broilers fed on T_4 (_α-lipoic acid) (1.88) followed by T_1_ (control) (1.85) and T_6_ (synthetic α-tocopherol and α-lipoic acid) (1.80) during experimental year 2011–12. The lowest FCR (1.66) was found in broilers fed on T_2_ (natural α-tocopherol). The broilers fed on wheat germ oil (natural α-tocopherol) gained maximum weight in both of the experimental years by consuming the same feed as compared to other treatments. The results of the present study are in consistent with the findings of [20] who reported that the highest FCR in the group with wheat germ enriched feed in rats. Rezaeipour et al. [32] found that feed conversion ratio improved linearly with increase of α-tocopherol in the feed of broilers. The results of the present are further supported by the findings of [33] who described that feed efficiency increased statistically with α-tocopherol supplementation. It is obvious from the results that feed containing α-lipoic acid supplementation alone yielded higher FCR because it gained less weight which is also confirmed by the findings of [27]. The present exploration indicated regarding FCR, the natural α-tocopherol showed better FCR as compared to control; conclusively α-lipoic acid supplemented feed exhibited an increasing trend in FCR.

### Antioxidant potential of broiler meat

#### Total phenolic contents in breast and leg meat

The total phenolic contents of broiler breast and leg meat for different feed treatments have shown in Table 2. The results indicated that total phenolic contents of breast meat was found to be in the range of 112.25±2.1 to 162.73±4.8 mg GAE/100 g and 111.89±2.5 to 162.18±4.5 mg GAE/100 g during the experimental years 2010–11 and 2011–12, respectively. The highest total phenolic contents (162.73±4.8 mg GAE/100 g meat) was recorded in broiler breast meat receiving T_5_ (natural α-tocopherol and α-lipoic acid) followed by T_6_ (synthetic α-tocopherol and α-lipoic acid) (149.33±4.1 mg GAE/100 g meat). However the lowest phenolic contents (112.25±2.1 mg GAE/100 g meat) was found in broilers fed on T_1_ (control) during the experimental year 2010–11. The trend of the total phenolic contents were found identical during the second experimental year.

**Table 2:** Total phenolic contents (mg GAE/100 g meat) in the breast and leg broiler meat

**Treatment**	**Breast meat**			**Leg meat**		
	**2010**	**2011**	**Means**	**2010**	**2011**	**Mean**
**T**_**1 **_**(control)**	112.25±2.1	111.89±2.5	112.07±2.8f	107.77±2.1	104.57±1.9	106.17±2.4f
**T**_**2 **_**(NAT)**	138.10±4.6	137.25±2.6	137.67±3.4c	125.86±2.6	126.68±2.8	126.27±2.1c
**T**_**3 **_**(SAT)**	122.60±3.4	122.10±2.1	122.35±2.7e	111.17±2.3	111.44±2.4	111.31±2.6e
**T**_**4 **_**(ALA)**	129.21±3.6	128.07±2.3	128.64±2.6d	122.46±2.2	122.73±2.6	122.60±2.9d
**T**_**5 **_**(ALA+NAT)**	162.73±4.8	162.18±4.5	162.46±3.7a	149.67±3.3	146.07±3.2	147.87±3.4a
**T**_**6 **_**(ALA+SAT)**	149.33±4.1	148.33±3.7	148.83±3.2b	136.54±3.5	133.48±3.8	135.01±3.9b
**Means**	135.70±3.6a	134.97±3.7a		125.58±2.9a	124.16±2.6a	

Values are mean ±S.D of three independent measurements.

Means sharing similar letter in a row or in a column are statistically non-significant (P>0.05).

NAT= Natural α-tocopherol, SAT= Synthetic α-tocopherol, ALA= α-Lipoic acid.

The total phenolic contents of broiler leg meat fed on different feed treatments ranged from 107.77±2.1 to 149.67±3.3 mg GAE/100 g meat and 104.57±1.9 to 146.07±3.2 mg GAE/100 g meat during the experimental years 2010–11 and 2011–12, respectively. The highest total phenolic contents (149.67±3.3 mg GAE/100 g meat) was found in broiler leg meat receiving T_5_ (natural α-tocopherol and α-lipoic acid) followed by T_6_ (synthetic α-tocopherol and α-lipoic acid) (136.54±3.5 mg GAE/100 g meat) during the experimental year 2010–11. In the same experimental year the lowest total phenolic contents (107.77±2.1 mg GAE/100 g meat) were found in T_1_ (control). The trend of the total phenolic contents were found identical during the second experimental year.

In the present study the breast meat contained higher total phenolic contents than that of the leg meat. This finding is in consistent with the results of [26] who reported that the treated-meat containing groups fed on α-lipoic acid and α-tocopherol supplemented feed yielded more phenolic content than those from the control group. The treated breast meat from the group fed 150 mg/g α-lipoic acid supplemented feed contained the highest level of total phenolics. The total phenolic content of treated breast meat higher than that of treated leg meat, suggesting that breast meat contains more antioxidants than leg meat. The phenolic antioxidants present in the broiler meat may be consumed to prevent oxidative damage, such as oxidative stress, occurring in the control. The antioxidant activity of α-lipoic acid is well known [34]. Therefore, α-lipoic acid may also be consumed to prevent this oxidative damage and, subsequently, to increase the amount of total phenolics in the treated meat compared with the control. The results of the present study are also consistent with the findings of [35,36] who found α-tocopherol possesses high antiradical power showed that total phenolic contents were higher in the antioxidants enriched treatments as compared to control.

The phenolic contents reflect the antioxidant activity of the meat tissue. Higher the total phenolic contents higher will be the free radical scavenging activity. The results of the present study are line with the findings of [7] who reported that the group fed on combination of α-lipoic acid and α-tocopherol supplemented feed exhibited more total phenolics than that broiler fed on control. The highest total phenolic compounds have been found in the broilers leg meat fed on T_5_ (combination of α-lipoic acid and natural α-tocopherol), which may be due to the fact that the phenolic compounds present in the wheat germ oil exerted antioxidant effect [37,38]. Some other scientists have also demonstrated the free radicals scavenging activity and strong antioxidant effect of phenolic compounds present in the wheat germ oil [39,40] which confirms the findings of the present study.

### Free radical scavenging activity (DPPH) of breast and leg meat

The free radical scavenging activity of broiler breast and leg meat fed on different feed treatments given in Table 3 indicated the DPPH free radical scavenging activity of breast meat varied from 75.39±0.9 to 85.28±1.6% and 75.88±1.1 to 84.84±1.5% during the experimental years 2010–11 and 2011–12, respectively (Table 3). The highest scavenging activity (85.28±1.6%) for free radical scavenging activity was found in broiler breast meat fed on T_5_ (natural α-tocopherol and α-lipoic acid) followed by T_6_ (synthetic α-tocopherol and α-lipoic acid) (83.23±1.4%). The lowest scavenging activity (75.39±0.9%) was recorded in broiler breast meat fed on T_1_ (control) during the experimental year 2010–11. The pattern of the free radical scavenging activity was found same during the repeat experiment. The DPPH free radical scavenging activity of leg meat was found to be in the range of 74.11±1.4 to 85.09±1.7% and 75.17±1.6 to 84.27±1.9% during the experimental years 2010–11 and 2011–12, respectively (Table 3). The highest inhibition (85.09±1.7%) for free radical scavenging was observed in T_5_ (natural α-tocopherol and α-lipoic acid) followed by T_6_ (synthetic α-tocopherol and α-lipoic acid) (82.95±1.1%) while the lowest free radical scavenging activity (74.11±1.4%) was exhibited in T_1_ (control) during the experimental year 2010–11. Similar pattern was exist during the repeat experiment for the free radical scavenging activity.

**Table 3:** Free radical scavenging activity (DPPH) (%) in broiler breast and leg meat

**Treatments**	**Breast meat**			**Leg meat**		
	**2010**	**2011**	**Means**	**2010**	**2011**	**Mean**
**T**_**1 **_**(control)**	75.39±0.9	75.88±1.1	75.63±1.3f	74.11±1.4g	75.17±1.6f	74.64±1.1f
**T**_**2 **_**(NAT)**	82.56±1.2	81.75±1.5	82.15±1.4c	81.55±1.6c	81.19±1.8c	81.37±1.5c
**T**_**3 **_**(SAT)**	79.32±1.1	78.71±1.3	79.01±1.3e	78.59±1.8e	79.55±1.4d	79.07±1.3e
**T**_**4 **_**(ALA)**	80.85±1.4	80.14±1.7	80.50±1.6d	80.11±1.6d	80.25±1.3d	80.18±1.8d
**T**_**5 **_**(ALA+NAT)**	85.28±1.6	84.84±1.5	85.06±1.6a	85.09±1.7a	84.27±1.9a	84.68±1.6a
**T**_**6 **_**(ALA+SAT)**	83.23±1.4	82.96±1.9	83.10±1.5b	82.95±1.1b	82.40±1.6b	82.68±1.3b
**Means**	81.10±1.8a	80.71±1.7a		80.40±1.5a	80.47±1.8a	

Values are mean ±S.D of three independent measurements.

Means sharing similar letter in a row or in a column are statistically non-significant (P>0.05).

NAT= Natural α-tocopherol, SAT= Synthetic α-tocopherol, ALA= α-Lipoic acid.

In the present study the breast meat possesses slightly more antioxidant activity than that of the leg meat which is in consistent with the findings of [26] who reported that breast meat showed stronger effects than those from leg meat that correlate to the total phenolic contents. These results were also consistent with reports of [36]. The present results are concurred with the [35] who reported α-tocopherol have high antiradical power. The results of the present study are inconsistent with the findings of [7] reported that the diet having group with combination of α-lipoic acid and α-tocopherol supplemented in the diet of broiler exhibited more antioxidant activity than the control. In the present results the highest antioxidant activity was found in the treatment combination of natural α-tocopherol and α-lipoic acid which is in agreement with the findings of Traber, (1999) whose results showed that natural α-tocopherol revealed 1.7-4 times the free radical scavenging strength than that of synthetic tocopherol. The meat from poultry fed with vitamin E supplemented feed has also exhibited a higher antioxidant activity than poultry fed normal feed [41]. α-Lipoic acid and α-tocopherol synergistically quench reactive oxygen species and subsequently inhibit the oxidative damage in biological systems [42]. It is obvious from the present results that the treatment with the combination of α-lipoic acid and wheat germ oil possess the highest antioxidant activity which is in line with the study of other scientists. The phenolic compounds found in wheat germ oil also have free radicals scavenging activity [37,38]. Some other scientists have also demonstrated the free radicals scavenging activity and strong antioxidant effect of phenolic compounds present in the wheat germ oil [39,40] which is confirmed in the present study.

### Ferric reducing antioxidant power in breast and leg meat

The ferric reducing antioxidant power of broiler breast and leg meat for different feed treatments is given in Table 4 and ranged between 586±12.5 to 655±13.4 μmol/Fe^+2^/g and 588±14.6 to 659±14.9 μmol/Fe^+2^/g during the experimental years 2010–11 and 2011–12, respectively (Table 4). The treatment T_5_ (natural α-tocopherol and α-lipoic acid) exhibited the highest ferric reducing antioxidant power (655±13.4 μmol/Fe^+2^/g meat) followed by T_6_ (synthetic α-tocopherol and α-lipoic acid) (627±14.9 μmol/Fe^+2^/g meat) and T_2_ (natural α-tocopherol) (617±11.6 μmol/Fe^+2^/g meat). The lowest ferric reducing antioxidant power (586±12.5 μmol/Fe^+2^/g meat) was yielded by broilers fed on T_1_ (control) during the experimental year 2010–11. The trend of ferric reducing antioxidant power of breast meat during experimental year 2011–12 was observed similar in repeat experiment. The ferric reducing antioxidant power of leg meat varied from 589±11.9 to 651±13.8 μmol/Fe^+2^/g meat and 591±13.2 to 645±13.8 μmol/Fe^+2^/g meat between the experimental years 2010–11 and 2011–12, respectively (Table 4). The broilers fed on T_5_ (natural α-tocopherol and α-lipoic acid) gave highest ferric reducing antioxidant power (651±13.8 μmol/Fe^+2^/g meat) was observed in followed by T_6_ (synthetic α-tocopherol and α-lipoic acid) (630±12.3 μmol/Fe^+2^/g meat) and. The broilers fed on T_1_ (control) possessed lowest ferric reducing antioxidant power (589±11.9 μmol/Fe^+2^/g meat) was found in) during the experimental year 2010–11. The trend with respect to ferric reducing antioxidant power was found identical for treatment during the repeat experiment.

**Table 4:** Ferric reducing antioxidant power (FRAP) of broiler breast and leg meat

**Treatments**	**Breast meat**			**Leg meat**		
	**2010**	**2011**	**Means**	**2010**	**2011**	**Mean**
**T**_**1 **_**(control)**	586±12.5	588±14.6	587±11.3d	589±11.9	591±13.2	590±13.2d
**T**_**2 **_**(NAT)**	617±11.6	614±13.8	616±13.6bc	615±13.6	620±13.3	618±13.6bc
**T**_**3 **_**(SAT)**	597±11.8	601±11.3	599±13.3cd	596±13.8	599±14.3	597±14.1d
**T**_**4 **_**(ALA)**	610±13.4	608±14.2	609±14.9c	606±14.3	609±13.9	608±12.9cd
**T**_**5 **_**(ALA+NAT)**	655±13.4	659±14.9	657±16.8a	651±13.8	645±13.8	648±14.6a
**T**_**6 **_**(ALA+SAT)**	627±14.9	631±13.8	629±14.3b	630±12.3	635±13.4	633±12.8ab
**Means**	615.3±14.6a	616.6±13.6a		614.5±15.6a	617.0±12.7a	

Values are mean ±S.D of three independent measurements.

Means sharing similar letter in a row or in a column are statistically non-significant (P>0.05).

NAT= Natural α-tocopherol, SAT= Synthetic α-tocopherol, ALA= α-Lipoic acid.

Jung et al. [43] found that the reducing power of the broiler breast meat fed on 0.5 and 1.0% mixture of gallic acid and linoleic acid was significantly greater than that of control. These results also suggest that breast meat samples from the broilers fed mixture of Gallic acid and linoleic acid were capable electron donors for neutralizing free radicals. The increase in ferric reducing antioxidant power due to feeding of α-tocopherol and α-lipoic acid may be attributed to their higher antioxidant potential as mixture of Gallic acid and linoleic acid exerted greater values of this parameter than the control.

According to [44] the antioxidative action of reductones is based on the breaking of free radical chains by the donation of hydrogen atom. Banerjee et al. [45] have reported that higher reducing power was observed in the natural antioxidants enriched meat as compared to synthetic antioxidant enriched meat. In this assay, the reductants present in the antioxidants enriched meat cause reduction of Fe^3+^ to the Fe^2+^ form. The reducing power of a compound is related to its electron-transfer ability; therefore, the reducing capacity of a compound may serve as a significant indicator of its potential antioxidant activity. Amarowicz et al. [46] observed a direct correlation between antioxidant activities and reducing power which have been shown to exert antioxidant action by breaking the free radical chain through donation of hydrogen atom. The birds fed on T_5_ (natural α-tocopherol and α-lipoic acid) might be due to the high antioxidative activity of the ingredients used in feed. This results in higher total phenolic contents, DPPH and Frap which is also supported by the findings of different scientists described in the proceeding section.

### Thiobarbituric acid reactive substance assay (TBARS)

The lipid peroxidation is one of the major mechanisms of quality deterioration in foods. It occurs when hydroxyl radicals attack fatty acid side chains of membrane phospholipids. Malondialdehyde (MDA) is a product of lipid peroxidation and TBARS, as indicated by the MDA concentration, serves as an oxidative damage index. The changes in quality attribute results deterioration in flavor, color, texture, and nutritive value of the food due to the production of toxic compounds [47].

### Thiobarbituric acid reactive substances (TBARS) in breast meat

**Figure 3:**
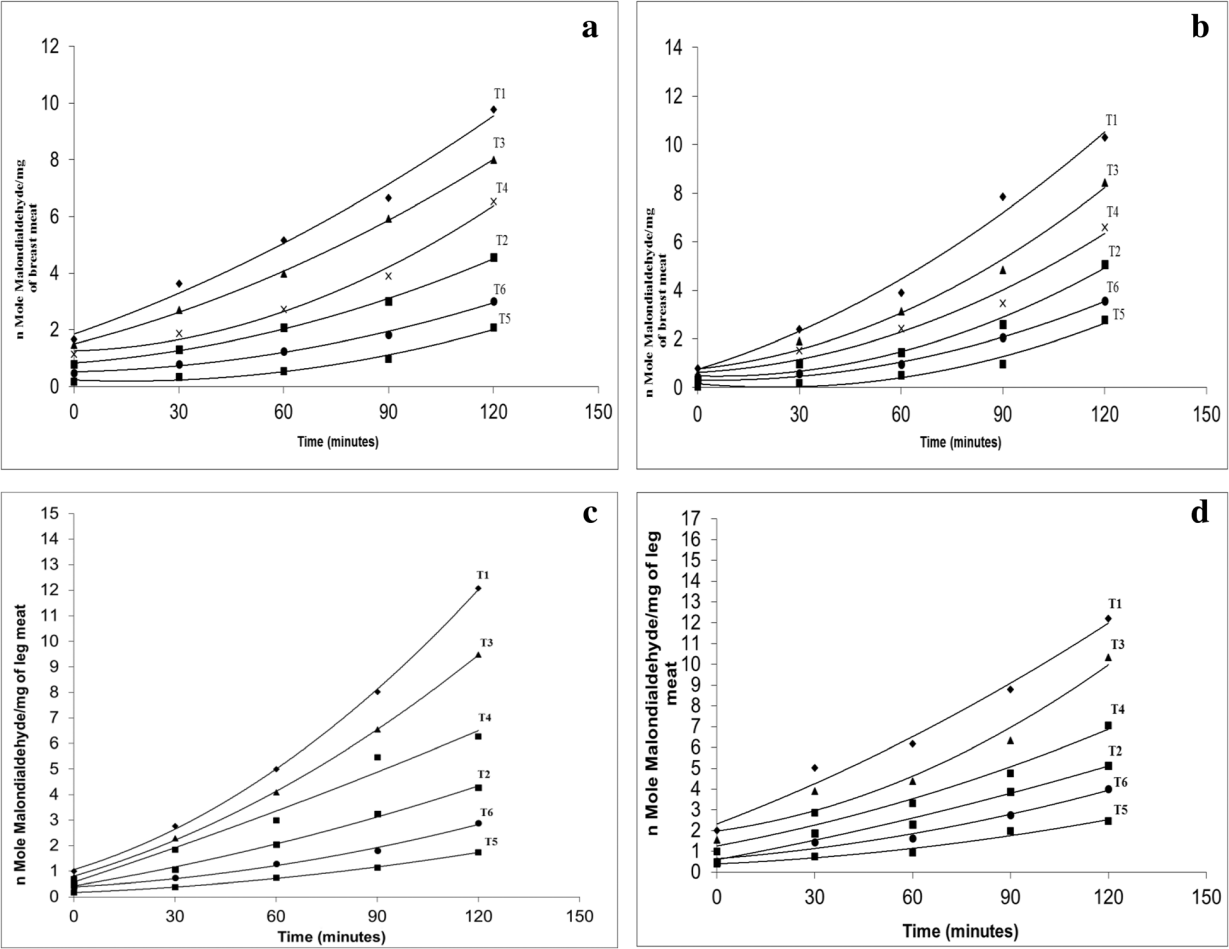
**Thiobarbituric acid reactive substances in breast and leg meat of broilers. A**: Thiobarbituric acid reactive substances (TBARS) reactive substances (TBARS). **B**: Thiobarbituric acid in breast meat of broilers during the experimental year 2010–11 in breast meat of broilers during the experimental year 2011–12. **C**: Thiobarbituric acid reactive substances (TBARS) in leg meat of broilers during the experimental year 2010–11. **D**: Thiobarbituric acid reactive substances (TBARS) in leg meat of broilers during the experimental year 2011–12.

The lipid peroxidation is the measure of malondialdehyde (MDA) compounds formed during auto-oxidation of lipids present in meat tissue. Higher malondialdehyde compounds concentration revealed higher amount of lipids which deteriorate the meat tissue. It is evident from the Figure 3a and 3b that the malondialdehydes produced in the breast meat of broilers at the beginning of the experiment (0 min) varied between (0.17 to 1.66 nmol malondialdehyde/mg meat and 0.06 to 0.80 nmol malondialdehyde/mg meat) during the experimental years 2010–11 and 2011–12, respectively. At the termination of the experiment (120 min) varied between (1.91 to 9.77 nmol malondialdehyde/mg meat and 2.78 to 10.29 nmol malondialdehyde/mg meat) during the experimental years 2010–11 and 2011–12, respectively. The breast meat of broilers fed on T_1_ (control) yielded significantly higher MDA (9.77 nmol malondialdehyde/mg meat) at the end of the experiment (120 min) followed by breast meat of broilers fed on T_3_ (synthetic α-tocopherol) (8.00 nmol malondialdehyde/mg meat) and T_4_ (α-lipoic acid) (6.27 nmol malondialdehyde/mg meat). The lowest MDA produced in the breast meat (1.91 nmol malondialdehyde/mg meat) was recorded in broilers fed on T_5_ (natural α-tocopherol and α-lipoic acid) during the experimental year 2010–11. The MDA produced in the breast meat during the repeat experiment was observed similar. The malondialdehydes produced in the leg meat of broilers at the initiation of the experiment (0 min) ranged between (0.17 to 1.00 nmol malondialdehyde/mg meat and 0.40 to 2.00 nmol malondialdehyde/mg meat) between the experimental years 2010-11 and 2011–12, respectively. However at the termination of the experiment (120 min) TBARS value ranged between (2.04 to 12.08 nmol malondialdehyde/mg meat and 2.78 to 12.20 nmol malondialdehyde/mg meat) during the experimental years 2010-11and 2011–12, respectively (Figure 3c and 3d). The leg meat of broilers fed on T_1_ (control) yielded significantly higher MDA (12.08 nmol malondialdehyde/mg meat) at the end of the experiment (120 min) followed by leg meat of broilers fed on T_3_ (synthetic α-tocopherol) (9.48 nmol malondialdehyde/mg meat) and T_4_ (α-lipoic acid) (6.52 nmol malondialdehyde/mg meat). The lowest MDA was recorded in the leg meat (2.04 nmol malondialdehyde/mg meat) of broilers fed on T_5_ (natural α-tocopherol and α-lipoic acid) during the experimental year 2010–11. The results for TBARS showed identical for the second experimental year.

It is also evident that the production of MDA in the breast meat increased significantly with increase in the time period. The results also showed that the breast meat of broilers enriched with the combination of natural α-tocopherol and α-lipoic acid (T_5_) feed. The lowest MDA concentration while the highest MDA concentration in the breast meat was found in the broilers fed on control (T_1_) in both of the experimental years. The trend is best explained by the polynomial curve. The results of the present study are in consistent with the previous studies of different scientist [10,24,48-50]. They all reported that the antioxidants supplemented in the feed of broilers increased the stability and decrease the MDA concentration in the broilers meat. Some other scientists found that α-lipoic acid supplemented in the feed of rats prevents the lipid peroxidation [51]. Similar results have been also found in the studies of [52]. Zulkhairi et al. [53] reported that TBARS levels were significantly lower in an α-lipoic acid treated group as compared to those in an untreated group, which confirms the results in the present study. In the present exploration, the TBARS value was lower in the broilers fed on antioxidants enriched treatments as compared to fed on control in both the experimental years this also will support by the many scientists and researchers as represented above.

Savitha et al. [54] reported that the supplementation of α-lipoic acid with other antioxidants decreased the levels of thiobarbituric acid reactive substances and improved the antioxidant status in skeletal muscles. The results of the present study showed that the MDA values were higher in the leg meat as compared to the breast meat which is supported by the findings of [7,26,55]. They reported that the MDA values found in leg meat were greater than those in breast meat, probably because of the greater fat content of the leg compared to the breast. The leg meat possesses a greater accumulation of poly unsaturated fatty acids as compared to breast meat. Same pattern has also been found in the findings of [56]. A possible reason may also be attributed that the leg muscle mainly comprised of slow twitch fibers has an increased oxygen consumption rate and higher level of ROS generation in comparison with the breast muscle mainly comprised of fast twitch fibers. In the present investigation, the TBARS value was lower in the antioxidants in meat raised through feeding antioxidants enriched treatments as compared to the control in both of the experimental years. The TBARS value was higher in the leg meat as compared to the control which is supported by many workers as explained above.

### Alpha-tocopherol content

The α-tocopherol is one of the major lipid soluble antioxidants belonging to tocopherol family which cannot be synthesized by the animal cells [57]. Alpha-tocopherol protects the membrane from oxidation. The supplementation of alpha-tocopherol in animals feed increases the deposition of lipoic acid as its concentration increases [58]. The level of vitamin E in the muscle depends on its dietary level, the duration of the supplementing period, the fiber type distribution and on metabolic characteristic. The absorption of vitamin E in broilers is variable with an average of 42% [59].

### Alpha-tocopherol content in the breast and leg of broiler meat

The results regarding α-tocopherol content of broiler breast and leg meat for different feed treatments given in Table 5 indicated that the α-tocopherol contents of breast meat varied significantly from 11.03±0.5 to 25.43±0.9 mg/g meat and 11.24±0.4 to 26.35±0.8 mg/g meat during the experimental years 2010–11 and 2011–12, respectively. The broilers fed on T_5_ (natural α-tocopherol and α-lipoic acid) showed the highest α-tocopherol content followed by T_6_ (synthetic α-tocopherol and α-lipoic acid) and T_2_ (natural α-tocopherol). The lowest α-tocopherol content was found in birds receiving feed T_1_ (control) during the experimental year 2010–11. The treatments behaved similarly during the repeat experiment. The α-tocopherol contents in leg meat significantly varied from 9.54±0.6 to 26.19±0.9 mg/g meat and 10.78±0.5 to 27.26±0.8 mg/g meat during the experimental years 2010–11 and 2011–12, respectively. The highest α-tocopherol content was observed in birds fed on T_5_ (natural α-tocopherol and α-lipoic acid) followed by T_6_ (synthetic α-tocopherol and α-lipoic acid) (23.66±0.8 mg/g meat) and T_2_ (natural α-tocopherol) (21.64±0.3 mg/g meat). The leg meat of birds fed on T_1_ (control) contained minimum content of α-tocopherol during the experimental year 2010–11. The behavior of deposition of α-tocopherol in leg meat of different feed treatments was also found similar during the repeat experiment.

**Table 5:** Alpha-tocopherol content (mg/g) in the broiler breast and leg meat

**Treatments**	**Breast meat**			**Leg meat**		
	**2010**	**2011**	**Mean**	**2010**	**2011**	**Mean**
**T**_**1 **_**(control)**	11.03±0.5	11.24±0.4	11.14±0.7f	9.54±0.6	10.78±0.5	10.16±0.4f
**T**_**2 **_**(NAT)**	21.29±0.9	22.44±0.9	21.86±0.5c	21.64±0.3	23.32±0.4	22.48±0.6c
**T**_**3 **_**(SAT)**	17.35±0.7	19.39±0.6	18.37±0.3d	18.54±0.4	19.34±0.3	18.94±0.7d
**T**_**4 **_**(ALA)**	13.77±0.5	12.86±0.4	13.31±0.7e	12.52±0.6	11.30±0.4	11.91±0.3e
**T**_**5 **_**(ALA+NAT)**	25.43±0.9	26.35±0.8	25.89±0.9a	26.19±0.9	27.26±0.8	26.73±0.8a
**T**_**6 **_**(ALA+SAT)**	23.82±0.8	24.21±0.5	24.02±0.6b	23.66±0.8	23.51±0.6	23.59±0.5b
**Means**	8.78±0.8a	19.41±0.5a		18.68±0.4a	19.25±0.7a	

Values are mean ±S.D of three independent measurements.

Means sharing similar letter in a row or in a column are statistically non-significant (P>0.05).

NAT= Natural α-tocopherol, SAT= Synthetic α-tocopherol, ALA= α-Lipoic acid.

It is evident from the results of the present study that α-lipoic acid in meat increased as α-tocopherol increased in feed and is agreement with [60,61] who reported that α-lipoic acid and α-tocopherol has synergistic effect with each other. The α-Lipoic acid requires at least 2 metabolic intermediate steps (via ubiquinol and ascorbate) to regenerate α-tocopherol. There is growing evidence that α-lipoic acid may act indirectly to maintain cellular antioxidant status by enhancing the synthesis of non-enzymatic antioxidants such as reduced glutathione, vitamin C and α-tocopherol (vitamin E) [62]. These studies showed that α-lipoic acid increases as α-tocopherol level increases in the feed. Some recent research on the supplementation of α-lipoic acid and α-tocopherol in the feed of broilers indicated that the deposition of α-tocopherol in meat increased with the presence of α-lipoic acid in the feed treatment which showed that there is a synergistic effect between the α-lipoic acid and α-tocopherol [7,26]. The results in the present study also showed that the deposition of the antioxidants was higher in the broiler fed on the combination of the antioxidants supplemented feed which is well supported by the scientists as discussed above.

The inclusion of α-lipoic acid into the diet caused a significant dose-dependent response in terms of leg muscle α-tocopherol level which may be due to the suppression of α-tocopherol consumption probably through the enhanced ROS scavenging by α-lipoic acid as well as due to recycling α-tocopherol by regenerating ubiquinol, vitamin C, and GSH during the redox cycles of α-lipoic acid and dihydrolipoic acid [63]. The present investigation showed that quantification of antioxidants was higher in the treatment where the combination of natural α-tocopherol and α-lipoic acid supplemented in the feed of broilers as compared to other treatments.

### Alpha-lipoic acid content

The alpha-lipoic acid is a novel antioxidant which has lipid lowering effect and protects the membranes of the meat tissue. The administration of alpha-lipoic acid through feed decreases oxidative stress and restore reduce levels of other antioxidants *in vivo*[4].

### Alpha-lipoic acid content in the breast and leg of broiler meat

The α-lipoic acid content of broiler breast and leg meat for different feed treatments described in Table 6 showed that α-lipoic acid contents in breast meat significantly varied from 6.70±0.3 to 41.31±1.2 mg/g meat and 7.86±0.4 to 42.13±1.4 mg/g meat during the experimental years 2010–11 and 2011–12, respectively. The highest α-lipoic acid contents (41.31±1.2 mg/g meat) was observed in birds fed on T_5_ (natural α-tocopherol and α-lipoic acid) followed by T_6_ (synthetic α-tocopherol and α-lipoic acid) (37.22±1.3 mg/g meat) and T_4_ (α-lipoic acid) (33.94±0.9 mg/g meat). The breast meat fed on T_1_ (control) contained minimum content of α-lipoic acid during the experimental year 2010–11. The behavior of deposition of α-lipoic acid in breast meat of different treatments was also found similar during the repeat experiment. The α-lipoic acid content of leg meat significantly varied from 8.25±0.7 to 42.50±1.6 mg/g meat and 7.59±0.5 to 43.06±1.2 mg/g meat during the experimental years 2010–11 and 2011–12, respectively. The broilers fed on T_5_ (natural α-tocopherol and α-lipoic acid) yielded significantly the highest α-lipoic acid content followed by T_6_ (synthetic α-tocopherol and α-lipoic acid). The leg meat of birds fed on T_1_ (control) possessed minimum content of α-lipoic acid during the experimental year 2010–11. The behavior of deposition of α-lipoic acid in leg meat of broilers fed on different feed treatments was also found similar during the second experiment.

**Table 6:** Alpha-lipoic acid contents (mg/g) in the broiler breast and leg meat

**Treatments**	**Breast meat**			**Leg meat**		
	**2010**	**2011**	**Means**	**2010**	**2011**	**Mean**
**T**_**1 **_**(control)**	6.70±0.3	7.86±0.4	7.28±0.5f	8.25±0.7	7.59±0.5	7.92±0.3e
**T**_**2 **_**(NAT)**	17.42±0.5	17.08±0.7	17.25±0.8d	16.89±0.6	15.29±0.8	16.09±0.8d
**T**_**3 **_**(SAT)**	13.23±0.7	14.81±0.6	14.02±0.6e	14.44±0.5	15.80±0.6	15.12±0.6d
**T**_**4 **_**(ALA)**	33.94±0.9	33.21±1.2	33.58±1.1c	32.97±1.1	34.00±1.4	33.48±1.2c
**T**_**5 **_**(ALA+NAT)**	41.31±1.2	42.13±1.4	41.72±1.5a	42.50±1.6	43.06±1.2	42.78±1.6a
**T**_**6 **_**(ALA+SAT)**	37.22±1.3	38.86±1.3	38.04±1.3b	38.75±1.4	41.30±1.3	40.03±1.2b
**Means**	24.97±0.9a	25.66±0.4a		25.63±0.6a	26.17±0.7a	

Values are mean ±S.D of three independent measurements.

Means sharing similar letter in a row or in a column are statistically non-significant (P>0.05).

NAT= Natural α-tocopherol, SAT= Synthetic α-tocopherol, ALA= α-Lipoic acid.

Some recent work on the supplementation of α-lipoic acid and α-tocopherol in the feed of broilers indicated that the deposition of α- lipoic acid increased with the presence of α-tocopherol in the feed treatment which showed that there is a synergistic effect between the α-lipoic acid and α-tocopherol [7,26]. The results in the present study also showed that the deposition of antioxidants was higher in the broiler meat give feed supplemented with combination of the antioxidants which is well supported by the scientists. Sohaib et al. [27] reported that maximum deposition of α-lipoic acid was found in the treatment with 150 mg/kg of feed than the other treatments which is in line with the findings of the present study.

## Conclusions

α-Lipoic acid and α-tocopherol and wheat germ oil significantly influenced the antioxidant status of the broiler meat. Wheat germ oil which was used as a source of natural α-tocopherol showed excellent results for the growth parameters as well as the antioxidant parameters of the broiler meat. Wheat germ oil was first time studied in the feed of broilers and showed excellent results. In the nutshell the combination of natural and synthetic antioxidants were more helpful in increasing the stability as well as quality of the broiler meat which has beneficial consequences for the human diet because poultry meat are widely consumed.

## Competing interests

The authors have no conflicts of interest. The authors alone are responsible for the content and writing of this manuscript.

## Authors’ contributions

The contribution of the each author for this paper was as follows, MSA carried out the trial of the broiler birds and also collected all the data of the trial. He also conducted all the analysis and drafted the manuscript. FMA helped in planning the experimental design and helped out during the research work. MIK, MS and SA provides technical assistance during the research work. MIK and MS provides technical assistance during research of the broiler and also guided in the analysis and statistical design of research trial. It is also confirmed that all the authors read and approved the final manuscript.
